# Validity and Reliability of an Inertial Sensor Device for Specific Running Patterns in Soccer

**DOI:** 10.3390/s21217255

**Published:** 2021-10-31

**Authors:** Guglielmo Pillitteri, Ewan Thomas, Giuseppe Battaglia, Giovanni Angelo Navarra, Antonino Scardina, Viviana Gammino, Dario Ricchiari, Marianna Bellafiore

**Affiliations:** 1 Sport and Exercise Research Unit, Department of Psychology, Educational Science and Human Movement, University of Palermo, 90144 Palermo, Italy; Guglielmo.pillitteri@unipa.it (G.P.); giuseppe.battaglia@unipa.it (G.B.); giovanniangelo.navarra@gmail.com (G.A.N.); ninoscardina93@msn.com (A.S.); viviana.gammino@gmail.com (V.G.); marianna.bellafiore@unipa.it (M.B.); 2 SSD Academy Ribolla Calcio, 90135 Palermo, Italy; ricchiaridario@libero.it

**Keywords:** inertial sensor, GPS, soccer, data tracking, performance monitoring

## Abstract

Electronic performance tracking devices are largely employed in team sports to monitor performance and improve training. To date, global positioning system (GPS) based devices are those mainly used in soccer training. The aim of this study was to analyse the validity and reliability of the inertial sensor device (ISD) in monitoring distance and speed in a soccer-specific circuit and how their performance compare to a GPS system. 44 young male soccer players (age: 14.9 ± 1.1, range 9–16, years, height: 1.65 ± 0.10 m, body mass: 56.3 ± 8.9 kg) playing in a non-professional soccer team in Italy, participated in the study. We assessed the players trough a soccer running sport-specific circuit. An ISD and a GPS were used to assess distance and speed. Data was compared to a video reference system, and the difference were quantified by means of the root mean square error (RMSE). Significant differences were found for both GPS and ISD devices for distance and speed. However, lower error for distance (dRMSE 2.23 ± 1.01 m and 5.75 ± 1.50 m, respectively) and speed (sRMSE 0.588 ± 0.152 m·s^–1^ and 1.30 ± 0.422 m·s^–1^, respectively) were attained by the ISD compared to the GPS. Overall, our results revealed a statistically significant difference between systems in data monitoring for either distance and speed. However, results of this study showed that a smaller error was obtained with the ISD than the GPS device. Despite caution is warranted within the interpretation of these results, we observed a better practical applicability of the ISD due to its small size, lower cost and the possibility to use the device indoor.

## 1. Introduction

In the last decade, electronic performance tracking devices have been widely employed to assess player’s external load (EL) in team sports [1,2,3,4]. Monitoring the physical and tactical behaviour of both training and competition is relevant to quantify and better understand the demands of competitions and develop specific training programs to improve performance and probably to decrease non-contact injuries risk [5,6,7]. To date, the most used devices to assess EL are semi-automatic multiple-camera video systems (VID), radio-based local positioning systems (LPS) and global positioning systems (GPS), sometime aided by triaxial accelerometers. In particular, in soccer, GPS devices are those mostly used for speed, metabolic demand and accelerations indicators [8,9,10,11,12,13,14,15,16,17].

The validity and reliability of such technology in soccer has been assessed by several investigations [18,19,20].However, it has been seen that these present some problems concerning their applicability and accuracy [4,21]. First, they must be used outdoors to receive an optimal signal [20,22]. In addition, the best accuracy can be only obtained with high-level devices. GPS that records at higher frequencies are more accurate than those sampling at lower frequencies. Indeed, Varey et al. reported that a 10 Hz GPS unit was more accurate for measuring instantaneous speed than a 5 Hz unit [13]. However, in recent years more reliable 20 or 50 Hz GPS devices are also available [10,23], although these are very expensive, and are usually employed only in elite sports. Another aspect concerning GPS is their relatively large size, which some people find not very comfortable to wear [24].

The physical demand of soccer players is approximately of 500 accelerations and decelerations per match [25,26]. Therefore, it is important to evaluate EL, that devices are able to detect such parameter. However, GPS-devices have been seen to possess lower accuracy for actions characterized by accelerations or change of directions [27].For example, Coutts et al. [28] showed that the GPS devices presented a satisfactory level of accuracy and reliability when referred to total distance and peak speeds during high-intensity, intermittent exercise, but were less reliable for high intensity activities as accelerations. 

In recent years, new technologies such as inertial sensors based on MicroElectroMechanical Systems (MEMS) have been developed, which have low production costs, are small in size and have the ability to measure kinematics over large periods of time [18,19,29,30]. Moreover, studies investigated the usefulness of these devices in different areas such as gait analysis [31,32], lower limb biomechanics [33], gait event detection [34], walking speed [35], movement sport specific [29] and sport performance [18,19,29,30]. MEMS can include triaxial accelerometers, triaxial gyroscopes, magnetometers and pressure sensors in a small size instrument. So, they are able to measure acceleration, including those induced by gravity, and angular velocity, among other parameters. Thus, integrated use of these sensors is described as Inertial Measurement Unit (IMU) or Inertial Sensors Devices (ISD). ISD have been employed in elite sport to better understand movement demands, particularly in indoor sports where GPS devices cannot be used [36]. It has to be noted that recent high-end GPS devices also integrate MEMS sensors [20], but these are just used to aid the GPS engine in case of temporary degradation or loss of signal, providing some orientation and state reference for the generated data [18,20]. The placement of the device on the athlete shoulder prevents the use of these MEMS for calculating precise motion data [20].

Some ISD can provide direct measures of instantaneous speed, distance and change of directions [18,19,29,30]. Therefore, these devices may help practitioners to better evaluate the demands of a sport (i.e., external load) according to commonly used training and performance assessment methods and to assist with physical training, injury prevention and technical analysis [2,3].

Despite this, ISD are still less used in professional team sports compared to GPS. Different studies have showed that they could properly monitor soccer performance [18,29]. However, these devices usually provide raw data or aggregated figures that are difficult to interpret and compare to classical metrics. Due to the novelty of these technologies, the accuracy of these new inertial tracker’s device still needs to be extensively characterized and validated.

One of the most common methods used to evaluate accuracy of devices in sports are the use of specific circuits, with known spatial measures. These have been widely used to evaluate, speed, acceleration in both training and competition [18,29]. Moreover, different studies have also shown that position may also be extracted using spatial coordinates [13,18,37]. In addition, in order to understand accuracy of a performance tracking device, it is necessary to use a reference video standard [18]. ISD commercially available devices possess similar characteristics between each other, since they are worn on the lower leg and provide several high-level data referred to the player movement instead of simple raw acceleration and rotational data [18,19,29,30]. This study was focused on to the first two parameters (i.e., speed and distance) since they are the most relevant in determining the external load, so a greater level of accuracy is generally required. Moreover, other external load indicators derived by speed and distance. Also, few attempts to date have been made to broadly evaluate the agreement between different technologies in team sports.

Hence, the aim of this study was to assess the validity and reliability of an inertial tracker’s device in soccer players using a predefined running sport-specific circuit. Measures from this device were compared to that of a video reference system and to a GPS device.

## 2. Materials and Methods

### 2.1. Participant

44 young male soccer players (age: 14.9 ± 1.1, range 9–16, years, height: 1.65 ± 0.10 m, body mass: 56.3 ± 8.9 kg) playing in a non-professional soccer team in Italy, participated in the study. Prior to participation, all players received comprehensive verbal and written explanations of the study, which was conducted within a period of three consecutive days. Signed informed consent to wear GPS/ISD sensors and to participate in the collection of spatiotemporal tracking data was provided to both the players and their parents. Institutional board approval for the study was obtained from the Ethics Commission of the University of Palermo (AIFA CE 150109). Moreover, all performance data were anonymized. This study conformed to the recommendations of the Declaration of Helsinki.

### 2.2. Experimental Design

To determine the validity and reliability of ISD for measuring instantaneous speed and distance, participants were requested to perform a running sport-specific circuit, which will be described in detail in the subsequent paragraphs. The trial required the participant to achieve different types of running activities (i.e., sprint running, performing acceleration, deceleration and change of direction effort and running at constant speed). Running speed was recorded using video analysis. Each players wore both a GPS device and a ISD during the trial. Before starting the assessment, a professional soccer physical trainer explained and showed the circuit. Thus, each player performed the entire circuit before trial recording. Each player performed the test twice and the best result was used for analysis. All players were tested from 17:00 pm to 20:00 pm, in the same synthetic grass field, using soccer shoes, and with the same environmental conditions (warm sunny day ⁓27 °C). The circuit has been divided into sections, and average speed was derived. For each player, the average speed in the individual sections recorded by the ISD and GPS were compared with the video analysis reference system. Mean error and standard deviations were calculated for each player. The aggregated data provided the performance in terms of error on the instantaneous speed of the ISD and GPS devices.

### 2.3. GPS, ISD Devices and Reference System

During each trial, the players wore one GPS unit (Qstarz BT-Q1000EX, 10 Hz available at www.qstarz.com, last accessed on 25 October 2021) [9,38,39], positioned on the upper back in a special vest. The GPS device was started 15 min prior to the assessment to make available the acquisition of satellite signals. After GPS fix, the average number of satellites in view was 12, the average HDOP was 0.8. So a good signal was acquired [22]. All data was acquired through a dedicated software (LaGalaColli V: 8.6.4.3). 

At the same time each player wore a ISD device (TalentPlayers TPDev, firmware version 1.3). Among the commercially available devices, the TalentPlayers was chosen as it provides very similar data compared to traditional GPS systems (i.e., instantaneous speed and distance, change of directions and metabolic data) and it is already applied by various Italian soccer teams by young and adults’ players (https://talentplayers.com, last accessed on 25 October 2021). This ISD tracker is a small wearable device integrating a 6 degree-of-freedom MEMS inertial sensor, able to provide acceleration and rotational data along 3 orthogonal axes. It is designed to be wore on the lower leg, by means of an elastic band or a specifically designed shin guard. It acquires real-time acceleration and rotational data at a frequency of 100 Hz per channel. Data is analysed in real-time by the device microprocessor using proprietary algorithms, that take into account the cinematic and dynamic of the lower leg. In particular, according to the manufacturer, the device employs an integration algorithm to evaluate the speed in the frontal direction, based on an adaptive version of the zero velocity update algorithm (ZUPT) [40,41]. This approach seems to be more accurate on a wider range of speeds compared to others techniques, for example the ones based on step rate only [42]. The integration of the gyroscopic signals and the cinematic of the motion are instead used to evaluate the change of directions. So, the device is able to provide raw data in the form of velocity (m/s), acceleration (m/s^2^), direction (degrees) and metabolic power (W/kg). These data, after processing can estimate instantaneous speed, accelerations, sprints, distance, metabolic parameters as energy expenditure, work ratio, metabolic thresholds and distance expressed at specific metabolic outputs and change of directions referred to the athlete’s centre of mass. The output data are down sampled at a lower rate (10 Hz) and stored in the device’s memory. However, since error computation requires homogeneous data, i.e., at the same sampling rate, 10 Hz was chosen because available on both GPS and ISD. For signal alignment purposes also the video signal was resampled at 10 Hz to be consistent with data from the GPS and the ISD. The resampling process has been furthered detailed within the data extraction section. Recorded data are downloaded by a smartphone or tablet via a Bluetooth interface and uploaded to the TalentPlayer web platform for storage and subsequent analysis. 

The trial was also recorded using a high-resolution video camera based on the Sony IMX258 image sensor, capable of capturing HD video (1920 × 1080 pixels) at 30 Hz, positioned at a central point of the pitch and operated to follow the athlete movement along the track [43]. Video recordings were analysed using Kinovea motion analysis software [44,45] to extract time data and the average speed on each section.

### 2.4. Data Extraction

GPS data were extracted through a dedicated software (LaGalaColli V: 8.6.4.3) while ISD data were downloaded from the acquisition device by the TalentPlayer mobile app (software version is 1.0.7) and uploaded to the TalentPlayers cloud, containing the visualization and analysis tools. Video-analysis allowed to accurately extract the travel time intervals of the various circuit sections using Kinovea software (version 0.7.10). Therefore, knowing the length of each section, it was possible to calculate the average speed. All data have been exported to a Microsoft 365 Excel spreadsheet (version 2107). For each player and for each device we calculated average speed (m/s) on each section. The total distance was calculated by integrating the instantaneous speed over time. Data from the video analysis was down sampled to 10 Hz. The three data series were aligned by evaluating the maximum of the cross-correlation among the data series, as suggested by FIFA standard [46]. Then errors on the average speed on each of the 16 sections for the ISD and GPS were calculated, with reference to the video analysis data, and the error on total distance was evaluated with reference to the known (measured) track length. This was done for each of player.

### 2.5. Simulated Soccer Running Specific Circuit

**Figure 1:**
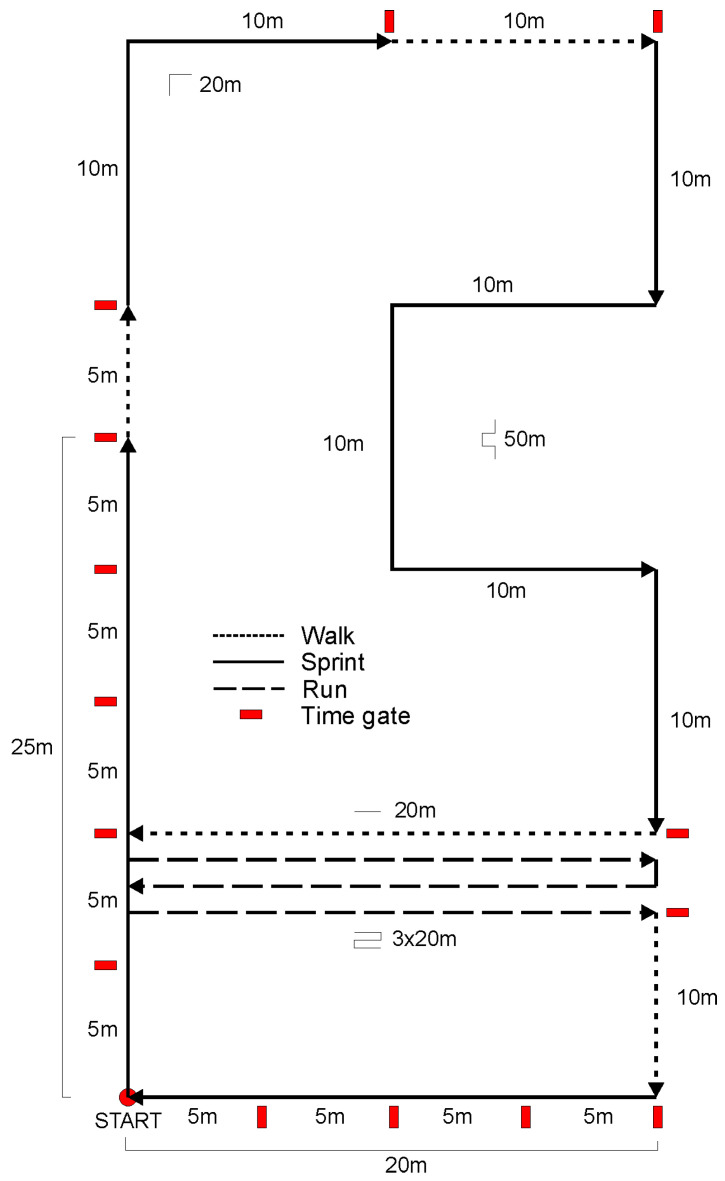
Circuit sport-specific running.

A predefined soccer running circuit with different running movement intensities (Figure 1) was used to analyse movements under controlled conditions. The circuit measured 220 m and included distinct elementary movement patterns:

(1). 25 m sprint + 5 m walk.
(2). 10 m sprint + 90° Change of direction (COD) right + 10 m sprint + 10 m walk.
(3). 10 m sprint + 90° COD right + 10 m sprint + 90° COD left + 10 m sprint + 90° COD left + 10 m sprint + 90° COD right + 10 m sprint.
(4). 20 m walk + 3 × 20 m shuttle (180°) running at constant speed + 10 m walk + 20 m sprint. Optical time gates for the video analysis were positioned at different points, dividing the circuit in 16 sections as showed in Figure 1. Each section is considered as the space between two consecutive time gates. These gates were used as optical marks for the video analysis to precisely measure the time spent by the subject in each section, and so to evaluate the average speed per section. These were used for analysis.

### 2.6. Statistical Analysis

The Statistical Package Jamovi (The Jamovi project (2021). Jamovi (Version 1.6) [Computer Software]. Retrieved from https://www.jamovi.org, last accessed on 25 October 2021) was used for statistical procedures and analyses. Accuracy of fundamental position data was estimated by means of the root mean square error (RMSE). Since we also analysed the error pertaining to speed measurements, we used sRMSE (m/s^–1^): instant speed root mean square error. To analyse the accuracy of fundamental and derived (instant speed) measures, a single sample *t*-test was conducted to determine if the mean of the resulting RMSEs of an individual GPS and ISD was statistically significantly different from zero. All measures significantly differed. A One-Way Manova with Tukey Post-Hoc test was conducted to establish differences between devices. Effect sizes (ES) were quantified to indicate the meaningfulness of the differences in the mean values. Cohen’s *d* effect sizes for the One-Way Manova was classified as trivial (0–0.19), small (0.20 ± 0.49), medium (0.50 ± 0.79) and large (>0.80) [47]. Descriptive statistics have been presented as the mean and standard deviation (SD). Lin’s concordance correlation between the reference system and ISD and between the reference system and GPS was also calculated. Statistical significance for all calculations was set at *p* < 0.05.

## 3. Results

### 3.1. Overview and Descriptive Data

**Figure 2:**
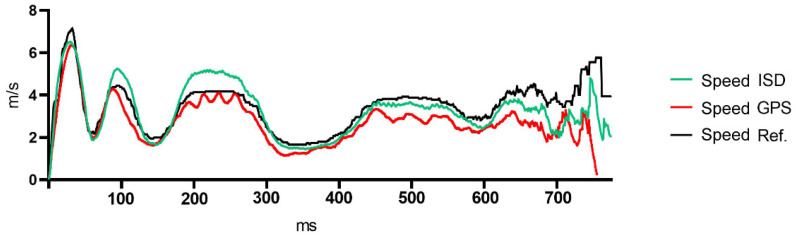
Aggregated data for ISD, GPS and reference system during soccer specific circuit. Accelerations and decelerations are observed during sprints and change of directions, respectively.

Table 1 reports descriptive data of GPS, ISD and video reference for speed and distance. Table 2 shows data of RMSE of speed and distance for both ISD and GPS. Lower error for distance (dRMSE) were attained by the ISD as compared to GPS (2.23 ± 1.01 m and 5.75 ± 1.50 m, respectively). Lower error for speed (sRMSE) was attained by the ISD as compared to GPS (0.588 ± 0.152 m·s^−1^ and 1.30 ± 0.422 m·s^−1^, respectively). Figure 2 shows aggregated data for speed assessed during the soccer specific circuit.

### 3.2. Distance

Table 2 shows measures for distance error dRMSE related to GPS and ISD. A significant difference between both GPS (*p* < 0.001) and ISD (*p* < 0.001) with reference video-analysis were observed F(2.86) = 9140, *p* < 0.001. A Tukey Post-Hoc test showed a significant difference between devices (*p* < 0.001). A very large effect size was found (d 28.21).

### 3.3. Speed

Table 2 shows measures of speed error sRMSE related to GPS and ISD. A significant difference between both GPS (*p* < 0.001) and ISD (*p* < 0.001) with reference video-analysis were observed, F(2.82) = 143, *p* < 0.001. A Tukey Post-Hoc test showed a significant difference between devices (*p* < 0.001). A large effect size was found (d 3.74).

### 3.4. Concordance between GPS and ISD Devices with Reference System

Lin’s concordance correlation was performed between the reference system and GPS and reference system and ISD devices. The GPS was less concordant (r = 0.183) to the video-analysis than the ISD (r = 0.801).

## 4. Discussion

The main aim of this study was to assess the accuracy of an inertial tracker device in soccer players using a predefined running sport-specific circuit and compare it with a more commonly used technology such as a GPS. The results of this investigation highlight that a statistically significant difference in error measure was present for both ISD and GPS compared to the reference system, however the ISD presented less error for both distance and speed and a higher concordance with the reference system compared to the GPS device. This result is in accordance with another study that assessed the measurement accuracy of the most used tracking technologies in professional team sports [18]. In this study VID, LPS and GPS were compared to a video reference system (VICON motion capture system), assessing measures and errors of distance, speed and acceleration on different sport-specific exercises. Also, in this case there was low accuracy for all devices when compared to the reference standard. Our study used a similar methodology to understand accuracy and reliability of the new inertial devices in comparison to the more common GPS. Another study compared the accuracy of stride time and stride length provided by an inertial sensor system and a reference system calculating RMSE [31]. The authors found good accuracy for such measures through the ISD. Despite the magnitude of errors for both tested devices did not comply with the reference system, these are acceptable in practical terms for sport monitoring. However, due to their relative novelty, validity of ISD should be further investigated in different context in team sports. To date, new sophisticated GPS also include accelerometers and gyroscopes, although these are usually employed in professional teams and are relatively expansive. In addition, GPS are usually bigger than ISD and present a poorer practical applicability since they can only be used outdoors. Conversely, ISD can also be used indoor without the need of coupling with external signals. Moreover, data sampling takes place differently. ISD measure real time movement through limb swing, while GPS use the Doppler effect of the satellite signals [18].

### 4.1. Distance

The results of the current study demonstrated that dRMSE were smaller in the ISD compared to the GPS. A study [18] reported that limited information on spatial accuracy about GPS are available, despite some authors analysed spatial motion behaviour [48,49] and determined distance metrics via differentiation of position data used [20]. Similar results were also retrieved by this study in which a significant difference was observed between all sensors and the reference system, with smaller errors in those with a higher sampling frequency. A greater GPS dRMSE was probably seen because of the loss of signal that the GPS may had undergone for a few seconds, time in which the speed tends to zero [20]. This explains why GPS has a good average speed performance, but a greater error on distance (underestimation) compared to the ISD.

### 4.2. Speed

Accurate assessment of speed, along with accelerations, can help to reveal important components of athletes high-intensity profiles in team sports. The results of the current study demonstrated that ISD was less accurate in measuring speed compared to the references system. However, as with distance, the sRMSE was smaller in the ISD compared to the GPS device. 

GPS determined speed by considering Doppler effect (i.e., rate of change in the satellites’ electromagnetic signal frequency). Despite studies revealed that using GPS Doppler measurements can provide useful speed accuracy [18], we found that ISD showed a smaller error (sRMSE) than GPS, resulting more accurate in speed detection. Many sports that used GPS or video-analysis, underestimated “real” physical demand when characterized by poor locomotor activities (i.e., jumping, collision, technical movements) [29]. Thus, ISD may be also used to detect movements and better understand EL in team sports. In perspective, significant uses may include injury-prevention strategies and return-to-play judgement.

### 4.3. Limitations

Our study assessed speed and distance measures during a sport specific circuit specifically designed, therefore it is still not possible to conclude if these results are still valid when the ISD device is used during match conditions (i.e., sport specific movement). Despite considerable statistical difference between measurement assessment was retrieved, these devices are still frequently employed during training, since the accuracy is considered to be good for practical applications. 

Small sided games [26] were not considered despite represent a crucial point on EL monitoring in soccer. Therefore, they should be considered in future studies. Data regarding other parameters were not taken into account in this study due to the technical assessment of each device. For example, it was not possible to study changes of direction accuracy since the tested technologies provided data which differ in nature and therefore, cannot be directly compared (i.e., the GPS provided a discrete count of direction changes only, while the ISD the instantaneous heading angle, average and maximum angle).

Moreover, although the circuit was accurately measured, the final distance covered by each player may have differed, since different strategies during change of directions may have been performed. A considerable amount of information can be derived by the instantaneous data provided by these devices, thus, the correct interpretation may require specialized knowledge.

## 5. Conclusions

In this study the performance of an ISD was assessed by using a reference system based on video analysis and a commonly used GPS device. Results of this study showed that there is a considerable difference data measurement between devices. However, a smaller error was observed in the ISD than the GPS device in the running soccer-specific circuit. Despite not having tested any sport specific movement, such as small sided games or official matches, the results suggests that the ISD could represent an alternative to GPS devices, especially considering the possibility to be used indoor. However, caution is needed when extending our results to other ISDs or to different sport specific circuits or populations. 

## Figures and Tables

**Table 1:** Descriptive measures for distance and speed.

	Video Ref.		GPS		ISD	
	Mean	SD	Mean	SD	Mean	SD
Estimated distance (m)	220	0	185	15.9	224	6.48
Estimated Speed (m·s^−1^)	4.41	0.30	3.65	0.45	4.26	0.35

Data are presented as means ± std.dv.

**Table 2:** Descriptive measures for distance error dRMSE related to GPS and ISD.

	GPS		ISD		MANOVA		
	Mean	SD	Mean	SD	*p*	ES	Diff Sign. Devices
dRMSE (m)	5.75	1.50	2.23	1.01	<0.001	VL	All
sRMSE (m·s^−1^)	1.30	0.422	0.58	0.152	<0.001	L	All

Data are presented as means ± std.dv; significant values *p* < 0.05; ES: Cohen’s *d*; L = Large; VL = Very large.

## Data Availability

Data available on reasonable request to the corresponding author.
